# Tests employed in the psychometric validation of the Insulin Treatment Appraisal Scale (ITAS) in T2DM patients; a systematic review of the literature

**DOI:** 10.1186/s41687-024-00792-y

**Published:** 2024-10-25

**Authors:** Saba Rasheed, Anees ur Rehman, Zermina Tasleem, Marryam Azeem, Muhammad Fawad Rasool, Arifa Mehreen, Saleh Karamah Al-Tamimi

**Affiliations:** 1 Department of Pharmacy Practice, Faculty of Pharmacy, Bahauddin Zakariya University, Multan, Pakistan; 2 Department of Political Science, Bahauddin Zakariya University, Multan, Pakistan; 3 Department of Zoology, Wildlife and Fisheries, University of Agriculture, Faisalabad, Pakistan; 4 Faculty of Pharmacy, University of Aden, Aden, Yemen

**Keywords:** Psychometric validation, ITAS, T2DM, Construct validity, Concurrent validity, Discriminant validity, Item-total correlation, Internal consistency reliability, Test-retest reliability, Systematic review

## Abstract

**Background:**

Psychological Insulin Resistance (PIR) and negative perceptions regarding insulin treatment are noteworthy challenges in T2DM management, which hinder the timely initiation of insulin treatment. To get past these obstacles a reliable tool is required to evaluate patients’ perspectives on insulin administration. Our study aims to conduct a comprehensive systematic review to evaluate the validity and reliability of different validation tests used in the psychometric validation of the ITAS in T2DM patients.

**Methods:**

A literature search was carried out, using PubMed, Google Scholar, EMBASE, Cochrane Library and Science Direct. Only those studies assessing content validity, construct validity, concurrent validity, discriminant validity, internal consistency reliability (Cronbach’ α), and items-total correlation were retrieved.

**Results:**

A total of 14 studies illustrated the validity and reliability of ITAS in T2DM patients. Content validity results of S-CVI was 0.97, and I-CVI was 0.8–1.00. Construct validity with factor loading was greater than the threshold value of 0.3. The concurrent validity of ITAS vs. PAID, WHO-5, and SPI was 0.35 (*P* < 0.05), −0.14 (*P* < 0.05), and 0.80 (*P* < 0.001) respectively. The mean difference between insulin and non-insulin group was significant (*P* < 0.001) showing reliable discriminant validity. Reported results of Cronbach’s α for the main scale (0.79–0.89), subscale-1 (0.72–0.9), and subscale-2 (0.61–0.89) showed “good to excellent” internal consistency reliability of ITAS. Item-total correlation results for the main scale, subscale-1, and subscale-2 were (0.40–0.82), (0.31–0.74) and (0.34–0.58) respectively. Test-retest reliability of ITAS was 0.571–0.87.

**Conclusions:**

Study findings confirm the robustness of various validation tests utilized in the psychometric validation of ITAS in T2DM patients. ITAS is a well-validated and reliable tool for determining the perspectives, PIR, and changes in patients’ perception over time and it can be used to overcome hurdles in the timely initiation of insulin treatment in T2DM patients.

## Background

Type-2 Diabetes Mellitus (T2DM) is a long-term metabolic condition marked by hyperglycemia due to decreased insulin production, insulin resistance, or both [1]. As per the International Diabetes Federation (IDF), diabetes incidence in 2019 was 463 million worldwide, which increased to 537 million in 2021 and is predicted to reach 643 million by 2030 [2]. Obesity, sedentary lifestyle, and poor nutritional diet are the major factors in the daily increase of T2DM cases worldwide [3]. In 2021, diabetes was responsible for 6.7 million deaths globally [4].

Oral hypoglycemic agents and insulin along with diet and physical exercise are the main treatment options in T2DM patients. However, relying on oral glucose-lowering agents alone is not a better option for managing persistently increased blood glucose levels. The patient’s body response to oral therapy decreases with time due to a decline in beta cell function [5]. Within 10 Years of DM, there is a 45–75% decline in pancreatic beta-cell function, which results in compromised response to oral drug therapy [6]. Later on, T2DM patients need insulin to achieve their targeted goals. After 6 years of diagnosis with T2DM, 50% of patients need insulin. Because oral glucose-lowering agents fail to manage the uncontrolled blood glucose level [7]. Insulin use has a various beneficial effect on patients’ quality of life. By using insulin, people can live a long and healthy life. They can do their usual routine physical activities. Insulin use causes an ultimate decrease in mortality [8].

Despite the benefits of insulin therapy, people often delay the prompt beginning of insulin administration. The most common factor in the delay of early initiation of insulin therapy is Psychological insulin resistance (PIR), a psychological state in which a patient experiences cognitive difficulties (negative perceptions) regarding aspects of insulin therapy [9]. PIR is a psychological disagreement towards the use of insulin in both patients (diabetic) and their prescribers [10]. Several factors are responsible for PIR including, the patient’s or doctor’s resistance to initiate insulin, diabetic social stigma, psychological burden, acceptance of alternative therapies, careful monitoring, time-consuming procedure to educate patients and their caretakers, injection fear, lifestyle restrictions, weight gain, adverse effects, and danger of hypoglycemia from insulin therapy, might cause a delay in starting insulin therapy [11–15]. There is a need for some strategies or tools to overcome these barriers and misconceptions regarding insulin use [16, 17].

Different types of questionnaires are used in diabetic patients to assess the patient’s perception regarding insulin treatment, diabetes-related emotional distress, and PIR and to identify the causes of resistance to initiating insulin therapy [18]. These questionnaires include Insulin Treatment Appraisal Scale (ITAS) [13], Problem Areas in Diabetes (PAID) [19], Type-2 Diabetes Stigma Assessment Scale (DSAS-2) [20], Diabetes Obstacles Questionnaire (DOQ) [21]. Among the above-mentioned questionnaires, ITAS is a validated and reliable diagnostic tool to assess the patient’s opinions about insulin treatment and to assess the patient’s perception during treatment. ITAS is a 20-items scale that consists of two sub-scales (16 negative statements and 4 positive statements). ITAS 20-items possible score ranges from 20 to 100 and subscale score ranges from 16 to 80 for 16-items of scale and 4–20 for 4-items of scale [5, 13].

ITAS was first developed for the Western population and then translated and validated in different languages and cultures. Different validation tests are employed in different studies to assess the validity of ITAS in different languages and cultures. While there is a substantial body of literature on the validation of ITAS, a critical examination reveals a notable gap in the absence of a comprehensive systematic review specifically focused on the tests employed in the psychometric validation of the ITAS. This necessitates a comprehensive systematic review of the psychometric validation studies of the ITAS in T2DM patients. Thus the objective of our study is to systematically review the ITAS to assess its validity and reliability in T2DM patients, as well as the validity and reliability of different validation tests employed in the psychometric validation studies of the ITAS in T2DM patients. In the future, this will lead to the development of more robust validation studies and can guide clinicians in selecting more appropriate treatment options. It will also help clinicians to overcome the challenges of PIR and negative perceptions regarding insulin treatment.

## Methods

### Study design

The PRISMA guidelines were adhered to conduct the systematic review [22]. The systematic review was enrolled in PROSPERO, with registration number CRD42023456774.

### Search strategy

Two evaluators separately searched the literature in PubMed, Google Scholar, EMBASE, Cochrane Library and Science Direct to search articles from 2007 (the first article published on ITAS) to September, 2023 (date last searched). We used different keywords such as Psychometric analysis, Insulin Treatment Appraisal Scale, ITAS, validity, reliability, psychological insulin resistance, perception and T2DM to search articles assessing the validity of ITAS. Furthermore, we also manually searched the reference list of the included articles.

### Inclusion and exclusion criteria

Two evaluators recruited only those studies for systematic review that fulfilled the following inclusion criteria: (1) well-diagnosed T2DM patients, (2) longitudinal observational studies, (3) studies assessing the validation of ITAS, (3) studies evaluating the reliability of ITAS, (4) studies reporting specific outcomes of interest (e.g. concurrent validity, construct validity, discriminant validity, internal consistency reliability, test-retest reliability), (5) original reports presenting primary research findings, (6) studies published in English language. All those studies were excluded from study that followed exclusion criteria: (1) studies including T1DM patients, (2) editorials, (3) conference abstracts, (4) peer reviews, (5) narrative reviews. In case of any problem or ambiguity, the two reviewers consult the third reviewer, who resolved the problem through discussion and consensus.

### Articles screening

Two evaluators separately screened the literature according to inclusion and exclusion standards. After title and abstract-based screening, related studies were enlisted. The search was limited to only English publications. Further included articles were screened based on full-text eligibility criteria. Any problem between two reviewers that occurred in the screening process was resolved through discussion or by the involvement of a third reviewer.

### Study selection

Two evaluators independently retrieved only original studies assessing desired study outcomes (concurrent validity, discriminant validity, construct validity, content validity, internal consistency reliability, and items-total correlation).

### Data extraction

After screening the articles, the two evaluators independently extracted the relevant information from the screened articles. The retrieved data included bibliographic information (author’s name, country, year), research methodology, research cohort, sample size, data source, research site, diagnostic parameters, study outcomes (concurrent validity, discriminant validity, construct validity, content validity, internal consistency reliability, and items-total correlation), demographic parameters (mean age) and clinical results (mean HbA_1C_, FBG). To verify the accuracy of the extracted the third reviewer compared the extracted data against the original study reports. Any disagreements and discrepancies in extracted data were resolved through discussion and consensus-building among the reviewers.

### Study outcomes

***Content validity*** assessment is used to examine whether the questions in the scale are applicable and appropriate to the measurement purpose and fully cover the topic for the target population. Content validity ranges from 0 to 1. Item-level Content Validity Index (I-CVI) ≥ 0.78, Scale-level Content Validity Index based on the universal agreement method (S-CVI/UA) ≥ 0.8, or Scale-level Content Validity Index based on the average method (S-CVI/Ave) ≥ 0.9 showed excellent content validity [23].

***Construct validity*** analyzes how well the test’s design-based hypotheses align with the findings of the use of the measure. Factor loading > 0.3 is considered a “moderate correlation” between the item and the factor [24].

***Concurrent validity*** is a correlation or comparison of a new scale with a validated scale called a criterion or gold standard. The scale should measure the same or similar construct. Pearson correlation coefficient value − 1 to + 1 showed a negative to positive correlation [25].

***Discriminant validity*** is employed to check whether the scale can differentiate between two different groups [26].

***Internal consistency reliability*** is used to measure the degree of interrelatedness among scale items. Cronbach’s α value ≥ 0.6 is considered “acceptable”, ≥ 0.7 is “good”, ≥ 0.8 is “strong” and ≥ 0.9 is “excellent” [27]. Guttmann’s λ2 value ≥ 0.70 − <0.90 is considered “reasonable” [28].

***Items total correlation*** assesses the reliability of multiscale items. For a good scale, the item-total correlation should range between 0.30 and 0.70 [29].

***Test-retest reliability*** is used to assess the consistent and reliable test results in the same group of patients over time. It falls between 0 and 1 and is classified as “poor” (< 0.5), “moderate” (0.5–0.75), “good” (0.75–0.9), and “excellent” (> 0.9) [30, 31].

## Quality appraisal

Two evaluators independently evaluated the quality appraisal of each included study using the “COSMIN Risk of Bias Checklist.” [32, 33]. The COSMIN Risk of Bias checklist assesses the quality of methodology and measurement properties such as content validity, structural validity, criterion validity, internal consistency, cross-cultural validity, responsiveness, reliability, hypothesis testing of construct validity, and measurement error. In this study, the assessment encompasses the quality of methodology as well as the quality of measurement properties, including internal consistency reliability, structural validity, and cross-cultural validity.

### Methodological quality assessment

Two evaluators separately conducted a methodical quality assessment of the ITAS internal structure, including internal consistency, structural validity, and cross-cultural validity. Every criterion in the box was given a 4-point rating system: very good, adequate, doubtful, and inadequate [32]. The “worst score count” principle of any standard in the box was used to determine the methodological quality. Any conflict between evaluators was resolved by the third evaluator through discussion.

### Measurement properties quality assessment

Two evaluators individually rated the quality of individual measurement property results according to the criteria for good measurement properties. Each criterion was rated as “ + ”, “ − ”, and “? ” indicating “sufficient”, “insufficient” and “indeterminate” respectively [34]. Any conflict between two evaluators in the quality assessment of measurement properties was resolved by the third evaluator through discussion.

### Evidence synthesis

For evidence synthesis qualitative summarized results of the ITAS’s structural validity, cross-cultural validity, and internal consistency reliability outcomes were used. Two evaluators separately overall rated the qualitatively summarized results about the standards for good measurement properties such as “sufficient (+)”, “insufficient (−)”, “inconsistent (±)”, and “indeterminate (?)” [34]. Then, two evaluators used a modified “GRADE” approach for grading evidence, i.e. “high”, “moderate”, “low”, and “very low” [34, 35]. The third reviewer verified the accuracy of the grading results and any disagreements were resolved through discussion and mutual agreement.

## Results

### Search results

**Fig. 1:**
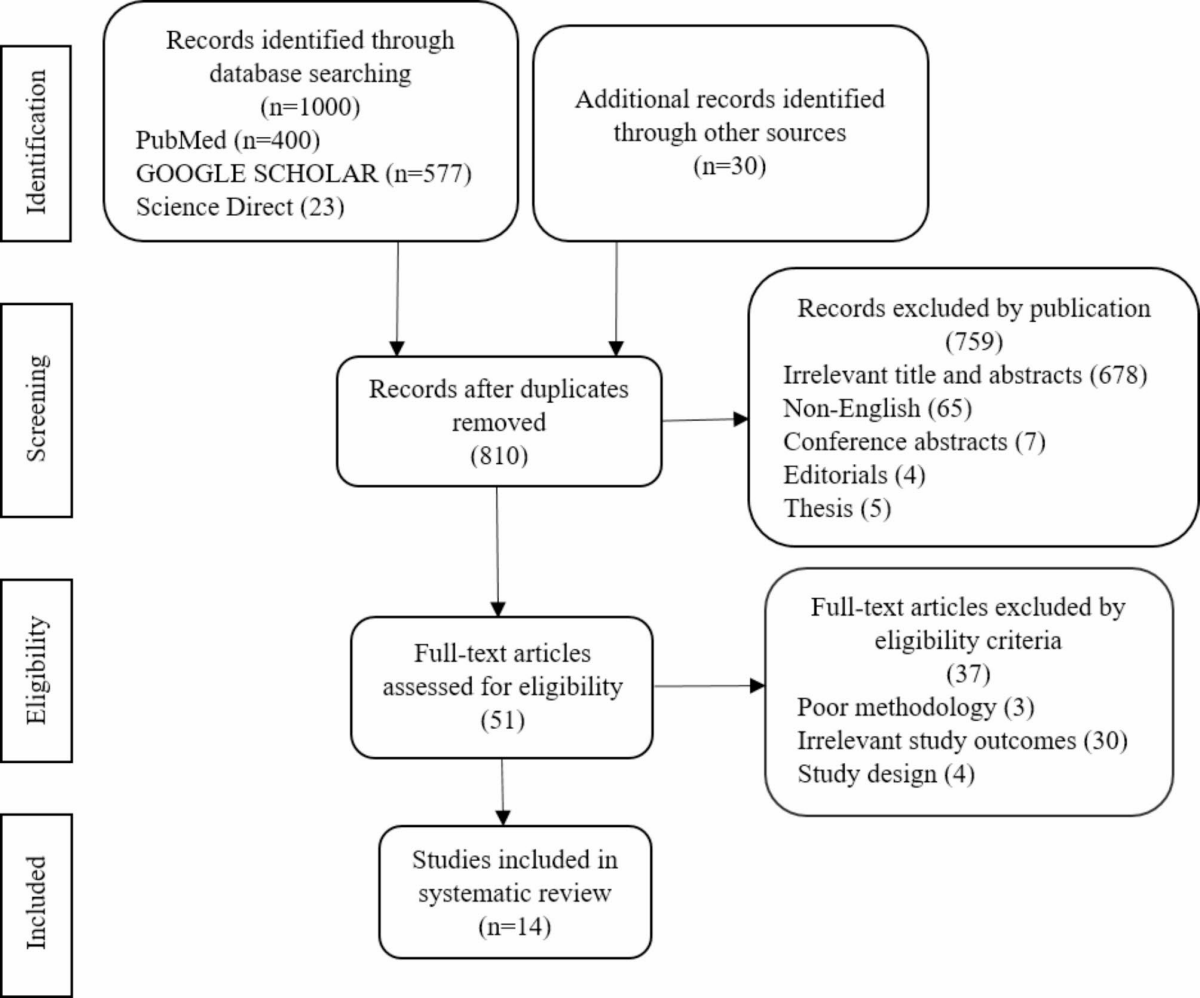
PRISMA flow diagram

Two reviewers identified 1000 records through database search engines, i.e. 577 in Google Scholar, 400 in PubMed, 23 in Science Direct, and 30 additional records identified through screening references. 810 records were obtained after removing duplicates. Of these, 759 articles were excluded due to irrelevant titles and abstracts (678), non-English (65), conference abstracts (7), editorials (4), and thesis (5). 51 articles were evaluated based on full-text eligibility criteria. 37 full-text articles were exempted due to poor methodology (3), irrelevant study outcomes (30), and study design (4). After the final evaluation, 14 eligible studies were incorporated into the systematic review. Figure 1 depicts the comprehensive study inclusion procedure.

### Characteristics of instrument and studies

Table 1 demonstrates the summary of studies that were part of the systematic review. Five of the included studies were conducted in Turkey, two each in the United States and Hong Kong, and one each in Australia, Romania, China, South Africa, and Cuba. All the studies were conducted in T2DM patients and the pooled sample size was 3,770 (range from 100 to 367). Patients were recruited from hospital settings, diabetes registration panels, and diabetic centers. In 10 studies, a self-administered ITAS questionnaire was administered, in 3 studies, an online ITAS questionnaire was filled out and in 1 study face-to-face interview technique was adopted. Patient’s clinical data included age ranged from 52.9 to 63Y, HbA_1C_ ranged from > 6.5–8.9%, and in only 1 study FBG 7.49 mg/dl was given.

**Table 1:** Characteristics of studies included in systematic review

Bibliographic information	Research methodology	Research cohort	Research site	Sample size	Data source	Scales	Diagnosis	Mean age (Y)	Mean HbA_1C_ (%)	Study outcomes
Snoek et al., US, 2007, [13]	Prospective	T2DM	HICIP registrants	282	Online survey	ITAS, PAID, WHO-5	• Physician diagnosed • HbA_1C_ test	59Y	6.80%	1. Construct validity (EFA) 2. Internal consistency reliability 3. Item-total correlation 4. Concurrent validity 5. Discriminant validity
Larkin et al., US, 2008, [36]	Prospective	T2DM	Hospital (Outpatient)	100	Online survey	ITAS, SPI	• CCI • HbA_1C_	62Y	7.10%	1. Internal consistency reliability 2. Concurrent validity
Makine et al., Turkey, 2009, [37]	Cross-sectional	T2DM	Hospital (Outpatient)	154	Self-administered	ITAS, PAID, CES-D	• HbA_1C_ test	57Y	6.70%	1. Internal consistency reliability
Holmes-Truscott et al., Australia, 2014, [28]	Cross-sectional	T2DM	NDSS registrants	748	Online Survey	ITAS	• Prescription based T2DM	59Y		1. Construct validity (EFA) 2. Internal consistency reliability 3. Item total correlation
Gherman, Romania, 2015, [38]	Prospective	T2DM	Diabetic centers	185	Self-administered	Romanian version of ITAS, BDI-2, SPB	• HbA_1C_ test	57Y	9%	1. Internal consistency reliability 2. Construct validity (EFA)
Lee, Hong Kong, 2016, [18]	Cross-sectional	T2DM	Hospital (Outpatient)	360	Self-administered	C-ITAS-HK	• WHO criteria • HbA_1C_ test	–	≤ 7%	1. Construct validity (EFA) 2. Internal consistency reliability 3. Test-retest reliability 4. Discriminant validity
Sürücü et al., Turkey, 2017, [39]	Methodological study	T2DM	Hospital (Outpatient)	367	Self-administered	Tr-ITAS	• Prescription based T2DM • HbA_1C_ test	52.9Y	8.90%	1. Construct validity (EFA) 2. Internal consistency reliability 3. Concurrent validity 4. Content validity 5. Discriminant validity
Lee, Hong Kong, 2018, [40]	Prospective	T2DM	Hospital (Outpatient)	200	Self-administered	C-ITAS-HK	• WHO criteria • HbA_1C_ test	63Y	7.20%	1. Internal consistency reliability 2. Construct validity (EFA) 3. Discriminant validity 4. Test–retest reliability 5. Content validity
Chen et al., China, 2020, [41]	Prospective	T2DM	Hospital (Outpatient)	200	Self-administered	C-ITAS	• Prescription based • HbA_1C_ test	60Y	8.30%	1. Internal consistency reliability 2. Construct validity (EFA) 3. Success rate 4. Content validity
Ngassa Piotie et al., South Africa, 2022, [42]	Cross-sectional	T2DM	Hospital (Outpatient)	253	Self-administered	Afrikaans version of ITAS	• HbA_1C_ test	57.9Y	9%	1. Construct validity (EFA) 2. Internal consistency reliability 3. Discriminant validity 4. Content validity
Hernández-García et al., Cuba, 2022, [3]	Cross-sectional	T2DM	Hospital (Outpatient)	198,	Self-administered	Spanish version of ITAS	• FBG test	57Y	7.47*	1. Construct validity (EFA) 2. Internal consistency reliability 3. Discriminant validity
İSMAİLOĞLU, Turkey, 2022, [43]	Prospective	T2DM	Hospital (ICU)	100	Self-administered	Tr-ITAS, DSMQ	• HbA_1C_ test	54Y		1. Internal consistency reliability
Özkan, Turkey, 2023, [7]	Cross-sectional	T2DM	Hospital (Outpatient)	293	Face-to-face interview	Tr-ITAS, CACMAS	• HbA_1C_ test • Prescription based T2DM	60Y	> 6.5%	1. Internal consistency reliability
Sağlam, Turkey, 2023, [44]	Cross-sectional	T2DM	Hospital (Outpatient)	330	Self- administered	Tr-ITAS, RPS-DM	• HbA_1C_ test	56Y	7.70%	1. Internal consistency reliability

*Note HICIP* Harris Interactive Chronic Illness Panel, *NIDSS* National Diabetes Services Scheme, *CCI* Charlson Comorbidity Index, *WHO* World Health Organization, *ICU* Intensive Care Unit, *FBG Fasting Blood Glucose in mg/dl, HbA_1C_ glycated hemoglobin, C-ITAS Chinese version of ITAS, Tr-ITAS Turkish version of ITAS, C-ITAS-HK Hong Kong modified version of ITAS, PAID Problem Areas In Diabetes, WHO-5 5-item World Health Organization Well-Being Index, CES-D Center for Epidemiological Studies-Depression Scale, DSMQ Diabetes Self-Management Questionnaire, BDI-2 Beck Depression Inventory-II, SPB Survey of Personal Beliefs, CACMAS Complementary, Alternative and Conventional Medicine Attitude Scale, SPI Self-Perceptions of attitudes toward Insulin treatment, RPS-DM Risk Perception Survey-Diabetes Mellitus

### Main results

Main results were categorized into main scale, subscale-1 (16-items negative appraisal) and subscale-2 (4-items positive appraisal). Tables 2 and 3, and 4 presented the results of the main scale, subscale-1, and subscale-2 respectively.

## Validity analysis

### Content validity

Content validity was assessed in 2 studies. It was assessed at the scale level and item level through S-CVI and I-CVI tests respectively. The reported value for the S-CVI test was 0.97. The reported value for the I-CVI test was between 0.8 and 1.00 [40, 41].

### Construct validity

Construct validity was assessed in 9 studies. EFA was used to evaluate the construct validity. In 7 studies, factors loading for all scale items was greater than the threshold value of 0.3 [3, 13, 14, 18, 28, 40, 41]. In 2 studies, factors Q1, Q2, Q7, Q9, Q12 and Q20 reported suppressing factor loading < 0.3 [38, 39].

### Concurrent validity

Concurrent validity was assessed in 2 studies. Items correlation of ITAS vs. PAID, WHO-5, and SPI scale was assessed using Pearson correlation coefficient. Concurrent validity was assessed in 1 study by correlating ITAS vs. PAID scale items and ITAS vs. WHO-5 scale items. In 1 study, it was assessed by correlation between ITAS vs. SPI scale items. The concurrent validity of ITAS vs. PAID, WHO-5, and SPI was 0.35 (*P* < 0.05), −0.14 (*P* < 0.05), and 0.80 (*P* < 0.001) respectively [13, 36].

### Discriminant validity

Discriminant validity was assessed in 5 studies. It was assessed at the main scale level and at subscale levels by comparing ITAS score among insulin and non-insulin treated patients. For main scale and both subscales the mean difference between insulin and non-insulin group was significant (*P* < 0.001). In insulin group the mean score rage form 26.28 to 49.79, whereas, in non-insulin group the mean score range from 37.36 to 61.60. For subscale 1, the mean score in insulin group range from 40.7 to 41.94, whereas, in non-insulin group the mean score range from 51.17 to 55.5. For subscale-2, in insulin group the mean score rage form 15.20 to 16.10, whereas, in non-insulin group the mean score range from 13.74 to 14.3. Discriminant validity was also assessed in 1 study by comparing ITAS score in insulin group with PIR and non-insulin group without PIR. For main scale and both subscales the mean difference between insulin group and non-insulin group was significant (*P* < 0.001). For main scale, the mean score in insulin group was 32.56 and in non-insulin group was 40.33. For subscale1, the mean score in insulin group was 47.09 and in non-insulin group was 53.78. For subscale 2, the mean score in insulin group was 14.49 and in non-insulin group was 13.29. PIR prevalence among insulin treated patients was from 6.8 to 29.4%, whereas, in non-insulin treated patients was from 44.9 to 63.1% [3, 13, 18, 40, 42].

**Table 2:** Validation results of main-scale reported in different studies

Reference	Construct validity	Concurrent validity	Discriminant validity	Content validity	Internal consistency reliability	Item-total correlation	Test-retest reliability	Success rate	Ceiling effect	Floor effect	Cut-off point	Effect size	ITAS score
Snoek et al., 2007, [13]	> 0.4	ITAS vs. PAID = 0.35 (*P* < 0.05) ITAS vs. WHO-5= -0.14 (*P* < 0.05)	NI = 61.6 Insulin = 48.8	NA	α = 0.89	NA	NA	NA	NA	NA	NA	NA	NI = 61.6 Insulin = 48.9
Larkin et al., 2008, [36]	NA	ITAS vs. SPI = 0.80 (*P* < 0.001)	NA	NA	α = 0.884	NA	NA	NA	NA	NA	NA	NA	35.8
Makine et al., 2009, [37]	NA	NA	NA	NA	IU α = 0.82 CU α = 0.87	NA	NA	NA	NA	NA	NA	NA	IU male = 52 CU male = 54 IU female = 56 CU female = 53
Holmes-Truscott et al., 2014, [28]	> 0.4	NA	NA	NA	Sample α = 0.87 NI α = 0.85 Insulin α = 0.84 Sample λ2 = 0.89 NI λ2 = 0.87 Insulin λ2 = 0.85	NA	NA	NA	NA	NA	NA	1.02 (*P* < 0.001)	Insulin = 50.2 NI = 60.7
Gherman, 2015, [38]	< 0.3	NA	NA	NA	α = 0.89	NA	NA	NA	NA	NA	NA	NA	53.8
Lee, 2016, [18]	> 0.3	NA	NI = 42.42 Insulin = 35.78(*P* < 0.001) PIR prevalence NI = 44.9% (95% CI, 39.4–50.4%) Insulin = 6.8% (95% CI, -0.64–14.24%)	NA	α = 0.78	NA	0.57 (*P* = 0.002)	NA	NA	NA	NA	NA	NA
Sürücü et al., 2017, [39]	< 0.3	NA	NA	NA	Sample α = 0.80 Insulin α = 0.81 NI α = 0.80 Sample λ2 = 0.80 Insulin λ2 = 0.84 NI λ2 = 0.60	0.40–0.82	NA	NA	NA	NA	NA	0.188 (*P* = 0.07)	Insulin = 41.05 NI = 39.41
Lee, 2018, [40]	> 0.4	NA	NI = 37.36 Insulin = 26.28 (*P* < 0.001) PIR(+ ve) = 40.33 PIR(-ve) = 32.56 (*P* < 0.001) PIR prevalence NI = 63.1% Insulin = 29.4%	I-CVI = 0.83-1.00 S-CVI = 0.97 Interrater reliability = 0.894 (95% CI 0.760–0.972)	α = 0.84	NA	0.87	NA	20%	NA	NA	NA	NA
Chen et al., 2020, [41]	> 0.4	NA	NA	≥ 80%	α = 0.72	NA	NA	IDV = 90% (18/20) ICV = 70% (14/20)	0%	0%	NA	NA	NA
Ngassa Piotie et al., 2022, [42]	> 0.4	NA	Insulin = 48.6 NI = 61.4 (*P* < 0.001)	Inter-rater reliability = ≥ 0.8	α = 0.85	NA	NA	NA	NA	NA	NA	1.24	NA
Hernández-García et al., 2022, [3]	> 0.4	NA	ITAS = 51.96 Insulin = 49.79 NI = 55.09 (*P* < 0.001)	NA	α = 0.747	NA	NA	NA	NA	NA	≥ 65	NA	NA
İSMAİLOĞLU, 2022, [43]	NA	NA	NA	NA	α = 0.8	NA	NA	NA	NA	NA	NA	NA	NA
Özkan, 2023, [7]	NA	NA	NA	NA	α = 0.78	NA	NA	NA	NA	NA	NA	NA	57.1
Sağlam, 2023, [44]	NA	NA	NA	NA	α = 0.89	NA	NA	NA	NA	NA	NA	NA	75.1

*Note NA* Not Available, *NI* Non-Insulin, *PIR* Psychological Insulin Resistance, *α* Cronbach’α, *λ2* Guttmann’ λ2, *CI* Confidence Interval, *IDV* Item-discriminant validity, *ICV* Item-convergent validity, *IU* Istanbul University, *CU* Cerrahpasa University

**Table 3:** Validation results of sub-scale 1 reported in different studies

Reference	Scale	Construct validity	Concurrent validity	Discriminant validity	Content validity & reliability	Internal consistency reliability	Item-total correlation	Test-retest reliability	Success rate	Ceiling effect	Floor effect	Cut-off point	Effect size	ITAS score
Snoek et al., 2007, [13]	Sub scale 1	NA	NA	NI = 55.5 Insulin = 44.1	NA	α = 0.9	0.46–0.74	NA	NA	NA	NA	NA	NA	NI = 55.5 Insulin = 44.1
Holmes-Truscott et al., 2014, [28]	Sub scale 1	NA	NA	NA	NA	Sample α = 0.90 NI α = 0.89 Insulin α = 0.85 Sample λ2 = 0.91 NI λ2 = 0.90 Insulin λ2 = 0.87	Insulin = 0.35-0.63 NI = 0.31–0.69	NA	NA	NA	NA	NA	1.04 (*P* < 0.001)	Insulin = 41.2 NI = 51.6
Gherman, 2015, [38]	Sub scale 1	NA	NA	NA	NA	α = 0.81	NA	NA	NA	NA	NA	NA	NA	43.68
Lee, 2016, [18]	Sub scale 1	NA	NA	NA	NA	α = 0.81	NA	NA	NA	NA	NA	NA	NA	NA
Sürücü et al., 2017, [39]	Sub scale 1	NA	NA	NA	NA	Sample α = 0.83 Insulin α = 0.64 NI α = 0.81 Sample λ2 = 0.84 Insulin λ2 = 0.64 NI λ2 = 0.83	NA	NA	NA	NA	NA	NA	0.21 (*P* = 0.89)	Insulin = 31.84 NI = 30.23
Lee, 2018, [40]	Sub scale 1	NA	NA	NI = 51.17 Insulin = 41.94 (*P* < 0.001) PIR(+ ve) = 53.78 PIR(-ve) = 47.09 (*P* < 0.001)	NA	α = 0.88	NA	0.78	NA	NA	NA	NA	NA	NA
Chen et al., 2020, [41]	Sub scale 1	NA	NA	NA	NA	α = 0.77	NA	NA	IDV = 88% (14/16) ICV = 63% (10/16)	NA	NA	NA	NA	NA
Ngassa Piotie et al., 2022, [42]	Sub scale 1	NA	NA	Insulin = 40.7 NI = 51.5 (*P* < 0.001)	NA	α = 0.84	NA	NA	NA	NA	NA	NA	1.17	NA
İSMAİLOĞLU, 2022, [43]	Sub scale 1	NA	NA	NA	NA	NA	NA	NA	NA	NA	NA	NA	NA	48.18
Özkan, 2023, [7]	Sub scale 1	NA	NA	NA	NA	NA	NA	NA	NA	NA	NA	NA	NA	47.3
Sağlam, 2023, [44]	Sub scale 1	NA	NA	NA	NA	α = 0.89	NA	NA	NA	NA	NA	NA	NA	64.1

*Note NA* Not Available, *NI* Non-Insulin, *PIR* Psychological Insulin Resistance, *α* Cronbach’α, *λ2* Guttmann’ λ2, *CI* Confidence Interval, *IDV* Item-discriminant validity, *ICV* Item-convergent validity

**Table 4:** Validation results of sub-scale 2 reported in different studies

Reference	Scale	Construct validity	Concurrent validity	Discriminant validity	Content validity & reliability	Internal consistency reliability	Item-total correlation	Test-retest reliability	Success rate	Ceiling effect	Floor effect	Cut-off point	Effect size	ITAS score
Snoek et al., 2007, [13]	Sub scale 2	NA	NA	NI = 14.3 Insulin = 15.2	NA	α = 0.68	0.34–0.53	NA	NA	NA	NA	NA	NA	NI = 14.3 Insulin = 15.2
Holmes-Truscott et al., 2014, [28]	Sub scale 2	NA	NA	NA	NA	Sample α = 0.69 NI α = 0.69 Insulin α = 0.68 Sample λ2 = 0.69 NI λ2 = 0.71 Insulin λ2 = 0.69	Insulin = 0.38-0.58 NI = 0.36–0.58	NA	NA	NA	NA	NA	0.04 (*P* = 0.58)	Insulin = 14.8 NI = 14.9
Gherman, 2015, [38]	Sub scale 2	NA	NA	NA	NA	α = 0.85	NA	NA	NA	NA	NA	NA	NA	10.13
Lee, 2016, [18]	Sub scale 2	NA	NA	NA	NA	α = 0.73	NA	NA	NA	NA	NA	NA	NA	NA
Sürücü et al.,2017, [39]	Sub scale 2	NA	NA	NA	NA	Sample α = 0.64 Insulin α = 0.64 NI α = 0.61 Sample λ2 = 0.64 Insulin λ2 = 0.64 NI λ2 = 0.83	NA	NA	NA	NA	NA	NA	0.014 (*P* = 0.04)	Insulin = 9.20 NI = 9.17
Lee, Hong Kong, 2018, [40]	Sub scale 2	NA	NA	NI = 13.74 Insulin = 15.64 (*P* < 0.001) PIR(+ ve) = 13.29 PIR(-ve) = 14.49 (*P* < 0.001)	NA	α = 0.61	NA	0.69	NA	NA	NA	NA	NA	NA
Chen et al., 2020, [41]	Sub scale 2	NA	NA	NA	NA	α = 0.76	NA	NA	IDV = 100% (4/4) ICV = 100% (4/4)	NA	NA	NA	NA	NA
Ngassa Piotie et al., 2022, [42]	Sub scale 2	NA	NA	Insulin = 16.1 NI = 14.0 (*P* < 0.001)	NA	α = 0.68	NA	NA	NA	NA	NA	NA	-0.8	NA
İSMAİLOĞLU, 2022, [43]	Sub scale 2	NA	NA	NA	NA	NA	NA	NA	NA	NA	NA	NA	NA	12.56
Özkan, 2023, [7]	Sub scale 2	NA	NA	NA	NA	NA	NA	NA	NA	NA	NA	NA	NA	14.1
Sağlam, 2023, [44]	Sub scale 2	NA	NA	NA	NA	α = 0.89	NA	NA	NA	NA	NA	NA	NA	11.1

*Note NA* Not Available, *NI* Non-Insulin, *PIR* Psychological Insulin Resistance, *α* Cronbach’α, *λ2* Guttmann’ λ2, *CI* Confidence Interval, *IDV* Item-discriminant validity, *ICV* Item-convergent validity

## Reliability analysis

### Content reliability

Content reliability was assessed in 2 studies. The content reliability of ITAS was assessed through inter-rater reliability using Kendall’s coefficient of concordance, and the test values were reported from 0.8 to 0.894 (95% CI (0.760–0.972) [40, 42].

### Internal consistency reliability

Reliability was evaluated in 14 studies. It was evaluated at the main scale and subscale level through Cronbach’ alpha and Guttmann’s λ2 tests. Cronbach’ alpha was used in 14 studies. For the main scale, internal consistency reliability score through Cronbach’s alpha test was reported from 0.74 to 0.89. For subscale-1, the reliability score was reported from 0.72 to 0.9. For subscale-2, the reliability score was reported from 0.61 to 0.89 [3, 7, 13, 18, 28, 36–44].

Guttmann’s λ2 was used in 2 studies. For the main scale, the internal consistency reliability score through Guttmann’s λ2 test was reported from 0.72 to 0.85. For subscale-1, the reliability score was reported from 0.73 to 0.88. For subscale-2, the reliability score was reported from 0.7 to 0.73 [28, 39].

### Item-total correlation

Item-total correlation was assessed in 3 studies. The item-total correlation was assessed at the main scale and subscale level. For the main scale, the item-total correlation score was reported from 0.40 to 0.82. For subscale-1, the item-total correlation score was reported from 0.31 to 0.74. For subscale-2, the item-total correlation score was reported from 0.34 to 0.58 [13, 28, 39].

### Test-retest reliability

Test-retest reliability was evaluated in 2 studies. It was evaluated at the main scale and both subscales level using test and retest scores’ Pearson’s correlation coefficient. For the main scale, the test-retest reliability score was reported from 0.571 to 0.87. For subscale-1, the test-retest reliability score was 0.78. For the subscale-2, the test-retest reliability score was 0.69 [18, 40].

### ITAS score

The ITAS score was given in 9 studies. The ITAS score was assessed in 3 studies for the main scale and both subscales among insulin and non-insulin treated patients. For the main scale, the ITAS sore was reported among insulin treated patients from 41.05 to 50.2 and in non-insulin treated patients from 35.8 to 61.60. For subscale-1, ITAS sore was reported among insulin treated patients from 31.84 to 48.18 and in non-insulin treated patients from 30.23 to 55.5. For subscale-2, ITAS sore was reported among insulin treated patients from 9.20 to 15.2 and non-insulin treated patients from 9.17 to 14.9 [13, 28, 39].

The ITAS score was given in 6 studies for the main scale and both subscales of the whole sample. For the main scale, the ITAS score was from 53.8 to 75.1. For subscale-1, the ITAS score was from 43.68 to 64.1. For subscale-2, ITAS score was from 10.13 to 14.1 [7, 36–38, 43, 44].

### Other psychometric analysis

The success rate was calculated in 1 study. The success rate was calculated at the main scale and subscale level by using item-total correlation of convergent validity and discriminant validity tests. For discriminant validity, the success rate was reported 90% for the main scale, 88% for the subscale-1, and 100% for the subscale-2. For convergent validity, the success rate was reported 70% for the main scale, 63% for subscale-1, and 100% for subscale-2 [41].

The cut-off point was evaluated in 1 study by A K-means cluster analysis. It was calculated using an ITAS score with a maximum negative perception score. The cut-off point ≥ 65 was established for inadequate perception regarding insulin treatment in T2DM patients [3].

The ceiling effect was assessed in 2 studies. 0% and 20% ceiling effects were calculated in each study [40, 41]. The 0% floor effect was also assessed in 1 study [41].

The effect size was calculated in 3 studies. The effect size was calculated at the main scale and subscale level for measuring the difference between groups through Cohen’s d test. For the main scale, the effect size value was reported between 0.188 and 1.24. For subscale-1, effect size was reported between 0.21 and 1.14 and for subscale-2, effect size was reported between − 0.8 and 0.04. Overall ITAS showed a “large” effect size which indicated the practical importance of research outcomes [28, 39, 42].

## Biasness results

Table 5 demonstrates the quality assessment of ITAS internal structure measurement properties. The structural validity and internal consistency reliability of ITAS showed consistent results and “high” quality evidence. The cross-cultural validity of ITAS showed inconsistent results.

**Table 5:** Quality assessment of studies

Reference	Assessment of methodological quality			Assessment of quality of measurement properties^a, b, c^		
	Structural validity	Reliability	Cross-cultural validity	Structural validity	Reliability	Cross-cultural validity
Snoek et al., 2007, [13]	Adequate	Very good	NA	−	−	
Larkin et al., 2008, [36]		Inadequate	NA		+	
Makine et al., 2009, [37]		Inadequate	Inadequate		+	?
Holmes-Truscott et al. 2014, [28]	Adequate	Very good	NA	+	−	
Gherman, 2015, [38]	Adequate	Very good	Doubtful	−	+	−
Lee, 2016, [18]	Adequate	Very good	Doubtful	?	+	+
Sürücü et al., 2017, [39]	Adequate	Very good	Very good	−	−	+
Lee, 2018, [40]	Adequate	Very good	Very good	−	−	−
Chen et al., 2020, [41]	Adequate	Very good	Very good	−	+	+
Ngassa Piotie et al., 2022, [42]	Adequate	Very good	Very good	+	−	+
Hernández-García et al., 2022, [3]	Adequate	Doubtful	Doubtful	?	+	+
İSMAİLOĞLU, 2022, [43]		Inadequate	Inadequate		+	?
Özkan, 2023, [7]		Inadequate	Doubtful		+	?
Sağlam, 2023, [44]		Very good	Doubtful		+	?

*Note NA* Not Applicable, Quality of Measurement properties score: ^a^ = Sufficient (+), ^b^ = Insufficient (−), ^c^ = Indeterminate (?)

## Discussion

Study results confirm the good psychometric properties of ITAS. ITAS is a well-validated and reliable instrument for determining the perspective of T2DM patients. The results of content validity show that the questions in the insulin treatment appraisal scale are appropriate, applicable, and comprehensive [39, 41]. Scale items are appropriate for measuring the patient’s perception of insulin treatment, and they also adequately describe the patient’s attitude and behavior toward insulin treatment. Factor loading > 0.3 shows a moderate correlation between the scale variable and factor, which means that the scale adequately measures the construct, and the study results show appropriate construct validity [13, 18, 42].

Cronbach’s alpha score of ITAS is “good to excellent” which shows the reliability of ITAS in T2DM patients [13, 40, 41]. No study reported internal consistency reliability values outside the threshold range. All items in the insulin treatment appraisal scale produce consistent and reliable results in T2DM patients of different populations. ITAS is a reliable tool for determining the patient’s perception toward insulin therapy. ITAS has good items-total correlation. For good psychometric properties of scale, the item-total correlation should be in range (0.3–0.7). In this review, study results reveal a good relationship between scale items and dimensions. The insulin treatment appraisal scale has a linear correlation with other scales in the expected direction and the linear association between scales is significant (*P* < 0.05) [13, 39, 42]. ITAS is a valid and reliable tool, as the study findings confirm “moderate to good” test-retest reliability of the ITAS main scale as well as subscale-1 and subscale-2 in T2DM patients. It produces consistent and reliable results in identical patient cohorts at two separate time points [18, 40].

The Insulin treatment appraisal scale effectively distinguishes between the perspectives of patients receiving insulin treatment and those who are not receiving it. This review study shows a high subscale-1 score (51.17–55.5 *P* < 0.001) and high PIR prevalence in non-insulin treated patients (44.9–63.1%), which indicates more negative perception towards insulin treatment [3, 13, 18, 40]. They believe that injecting insulin is painful, injecting insulin is embarrassing, and they are afraid of injecting with a needle. They also believe that taking insulin increases the risk of hypoglycemia and causes weight gain. These injection anxieties and misconceptions regarding insulin treatment are the major causes of more negative perceptions in non-insulin treated patients. Fear of injection and depression, which are more common in T2DM patients, are major factors of psychological insulin resistance [37, 42].

Insulin treated patients have high 4-item subscale-2 score (15.20–16.10 *P* < 0.001) and high PIR prevalence (6.8–29.4%), which indicates a more positive perception towards insulin treatment [7, 38]. They believe that taking insulin helps to prevent complications of diabetes, taking insulin helps to improve their health, taking insulin helps to maintain good control of blood glucose, and taking insulin helps to improve their energy level.

In the original study of ITAS, no cut-off score was given, but in 1 study cut-off point ≥ 65 was established for inadequate perception regarding insulin treatment in T2DM patients [3]. In future studies, this cut-off score can be used to identify the non-insulin treated patients with poor perception.

Biasness study of ITAS internal structure also shows “High” quality evidence for the structural validity and internal consistency of ITAS. The structural validity of ITAS has high-quality evidence with strong positive results and consistent findings in multiple studies of adequate methodological quality. The internal consistency of ITAS has high-quality evidence with strong positive results and consistent findings in multiple studies of adequate methodological quality. ITAS cross-cultural validity shows inconsistent results, and we can’t summarize these inconsistent results and grade the quality of the evidence. As we can see, studies involved in systematic review confirm good psychometric properties of ITAS.

Our study has several strengths. Our study is the first comprehensive systematic review specifically focused on validation studies of the ITAS, this targeted approach has enhanced the relevance and specificity of our findings. Our systematic review followed PRISMA guidelines, to ensure transparency in reporting methods and results, and allow reliability and validity of the review’s findings.

Our study has some limitations. It exclusively incorporates psychometric validation studies of the ITAS, with a potential for selection bias. In any of the included studies, the ITAS was not fully psychometrically validated, making it challenging to assess the robustness of the methods used. Only those research articles published in English can introduce language bias. Conference abstracts were excluded due to inadequate and unreliable information regarding psychometric properties.

Based on the findings of our systematic review, here are some potential areas for future research. Test interventions (e.g. counseling) aimed at improving insulin treatment appraisal in T2DM patients, using ITAS as an outcome measure. Based on the reliability findings focus on longitudinal studies to further explore its responsiveness and application in diverse populations, assessing its sensitivity to changes in patients’ beliefs and attitudes. Establish concurrent validity and identify areas for improvement by comparing with other scales assessing similar constructs. Examine the association between ITAS scores and clinical outcomes such as glycemic control, and treatment adherence. Investigate the psychometric properties of the ITAS in specific subgroups of T2DM patients such as elderly patients, adolescents, and comorbid patients, to understand its applicability.

## Conclusions

This systematic review affirms the robustness of various validation tests utilized in the psychometric validation of ITAS in T2DM patients. Study findings show ITAS is a well-validated and reliable instrument for assessing the attitude of T2DM patients towards insulin treatment and changes in patients’ perceptions over time. Notably, the findings support the ITAS suitability in assessing PIR and identifying and addressing challenges in the timely initiation of insulin in T2DM patients, leading to improved diabetes management and patient outcomes. By identifying specific fears and misconceptions, healthcare providers can use ITAS to create more personalized diabetes management plans and overcome barriers in the timely initiation of insulin treatment. This leads to better treatment adherence, improved glycemic control, and reduced complications, ultimately resulting in enhanced health outcomes and quality of life for patients with T2DM.

## Data Availability

Data will be available on request from the corresponding author.

## Abbreviations

DOQ: Diabetes Obstacles Questionnaire
DSAS-2: Type-2 Diabetes Stigma Assessment Scale
IDF: International Diabetes Federation
PAID: Problem Areas in Diabetes
PIR: Psychological Insulin Resistance
T1DM: Type 1 Diabetes Mellitus
T2DM: Type 2 Diabetes Mellitus
